# The Plasticity of the Antioxidant Defence System of Coastal Zooplankton Communities

**DOI:** 10.1002/ece3.73919

**Published:** 2026-06-28

**Authors:** Andriana Koutsandrea, Tytti‐Maria Uurasmaa, Katja Anttila, Jonna Engström‐Öst

**Affiliations:** ^1^ Åbo Akademi University Åbo Finland; ^2^ Novia University of Applied Sciences Ekenäs Finland; ^3^ Department of Biology University of Turku Turku Finland; ^4^ Tvärminne Zoological Station Hangö Finland

**Keywords:** antioxidants, eutrophication, hypoxia, oxidative stress, salinity, zooplankton community

## Abstract

Zooplankton are expected to enhance their antioxidant defences and activate stress‐related biomarkers in response to warming and reduced salinity or hypoxia and cyanobacteria blooms, reflecting both physiological adaptation to increased oxidative stress and a strategy to maintain cellular stability under changing environmental conditions. This study aims to assess how the antioxidant defence system and stress levels of the mesozooplankton community respond to varying environmental conditions in the changing Baltic Sea. We monitored biomarkers of oxidative stress (lipid peroxidation, LPX) and antioxidants (glutathione‐s‐transferase, GST and catalase, CAT) capacity of mesozooplankton in response to temperature, salinity, chlorophyll *a* and oxygen concentration at four locations in the northern Baltic Sea, during summer and autumn 2023. We show that antioxidants responded to different environmental conditions, whereas lipid peroxidation remained low and stable independent of environmental conditions, but showed a significant relationship with temperature at 10 m depth. Antioxidant responses in the mesozooplankton community varied across locations, influenced by both environmental conditions and mesozooplankton composition. GST activity was elevated in communities dominated by copepods and showed correlations with temperature, dissolved oxygen, and chlorophyll *a*. In contrast, CAT activity was higher in cladoceran‐rich assemblages and was significantly associated with salinity and dissolved oxygen. These findings indicate that environmental variables exert an influence on antioxidant enzyme activity, but species composition modulates the specific response patterns. Due to the plasticity of antioxidants, the oxidative damage remained low. The absence of detectable oxidative damage, even under suboptimal environmental conditions, suggests that the mesozooplankton community possesses a highly effective and adaptive antioxidant defence system.

## Graphical abstract

In this photo, a copepodite *Acartia* spp. is represented with *Vorticella* epiphytes. This photo was taken with the help of a PlanktoScope, which belongs to the Hellenic Centre for Marine Research. Photographer: Andriana Koutsandrea.

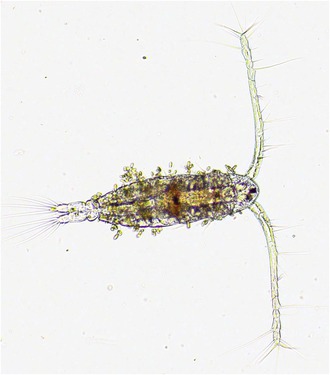

## 1 Introduction

If the concentration of CO_2_ keeps increasing in the atmosphere due to the burning of fossil fuels, warming will continue (Sanchez‐Lugo et al. 2019), and a large part of marine ecosystems will either change or go extinct (Mandal et al. 2022 and references therein). Global warming, eutrophication and associated processes, such as salinity change and oxygen deficiency, are serious threats that cause significant impacts on marine communities (Hoegh‐Guldberg and Bruno 2010; Kabel et al. 2012; Thompson et al. 2015; Raven and Beardall 2021). Thus, the oceans are becoming hostile environments for marine organisms and as a result, their populations may either decrease, or copepods can move to more suitable habitats, for example, polewards due to warming (Richardson 2008 and references therein; Villarino et al. 2015). Coastal eutrophication drives several changes in the system, among which acidification, hypoxia, and resulting decrease in biodiversity, are the most important (Kessouri et al. 2021). Due to warming and eutrophication, changes will be observed in phenological patterns, such as timing of migration, reproduction, biomass and abundance (Kessouri et al. 2021; Taipale et al. 2022; Forsblom et al. 2024). Eutrophication also affects the food quality and quantity of planktonic grazers (Suikkanen et al. 2013; Kuosa et al. 2017).

The Baltic Sea is a shallow brackish sea, hosting a mix of marine, freshwater and brackish‐water species (Leppäranta and Myrberg 2009; Viitasalo and Bonsdorff 2022). The surface salinity of the Baltic Sea is expected to decline, due to the mild winters, and increases in precipitation and freshwater run‐off (Holopainen et al. 2016 and references therein; Lehmann et al. 2022), which drive a decrease of marine zooplankton and an increase of freshwater species (Suikkanen et al. 2013; Hänninen et al. 2015; Kuosa et al. 2017). The Baltic Sea has also been severely impacted by eutrophication since the 1960s, and consequently, is subjected to large‐scale hypoxia (Conley et al. 2009; Andersen et al. 2017). The Baltic Sea is, furthermore, warming faster than other seas in the world, around 1°C per decade and its sub‐basins exhibit distinct responses to temperature rise (Lehmann et al. 2011; Kniebusch et al. 2019; Dutheil et al. 2022). Several papers reporting long‐term data have studied the relationship between warming and zooplankton; Suikkanen et al. (2013) showed that Baltic *Keratella* rotifers were positively related to elevated temperature, whereas predatory and suspension‐feeding crustaceans were favoured by cooler temperatures. Cladocerans and rotifers were strongly positively related to warming in the Gulf of Riga (Jansson et al. 2020). Zooplankton phenology was also shown to be affected by rising temperatures and earlier ice break‐up, for example, *Acartia* spp. appears earlier in the water column than previously (Forsblom et al. 2024).

In the Baltic Sea with the progressing warming and decline in salinity, marine taxa and large copepods such as *Centropages hamatus* , *Limnocalanus macrurus, Pseudocalanus elongatus* and *Temora longicornis* show decreasing abundances (Mäkinen et al. 2017). Further, these changes may force the marine species to oxygen‐deficient areas influencing their survival (Möller et al. 2015 and references therein). As zooplankton perform vertical migration to avoid predators, even under hypoxic conditions (Webster et al. 2015 and references therein) the changes in salinity and temperature could restructure the zooplankton community. Vuorinen et al. (1998) showed that the copepod/cladocera ratio decreased with the salinity in the Baltic Sea. Even though some brackish species can tolerate low salinity (e.g., *Acartia* spp. and *Bosmina* spp.; Hall and Lewandowska 2022), it is unknown whether their abundance will remain stable in the future, as these species, too, are facing multiple environmental stressors in the Baltic Sea.

Biomarkers can show how organisms respond to different biotic conditions, such as physical activity, growth, immune response, hormonal status, and predation risk (Costantini 2010), as well as to abiotic factors, such as temperature, salinity, and pH (Gorokhova et al. 2013; von Weissenberg, Mottola, et al. 2022; Lassoued et al. 2023). The increase in metabolism, due to elevated temperatures (Lushchak 2011), can cause oxidative damage in organisms if they do not have an efficient antioxidant defence system (Monaghan et al. 2009). Oxidative stress can occur when there is an imbalance between the antioxidants and reactive oxygen species (ROS). If the imbalance is significant, it can lead to damage in DNA, lipids, and proteins, or even mortality (Monaghan et al. 2009; Gorokhova et al. 2013). If the individual survives, oxidative stress could lead to effects on other biological processes (von Weissenberg, Jansson, et al. 2022). Previous work from the Baltic Sea shows that antioxidant glutathione‐s‐transferase (GST) activity increased in copepods during warming, salinity decrease, higher oxygen, and cyanobacteria blooms in both field and experimental settings (Vuori et al. 2015; Gorokhova and El‐Shehawy 2022; von Weissenberg, Mottola, et al. 2022; von Weissenberg, Jansson, et al. 2022). Also, the lipid peroxidation (LPX) increased in *Acartia* spp. at elevated temperatures and continued to rise during prolonged heatwaves and lower chlorophyll *a* (Glippa et al. 2018; von Weissenberg, Mottola, et al. 2022; von Weissenberg, Jansson, et al. 2022). Studies have also shown differences in the biomarker activities across species; in copepods (Vuori et al. 2015; Engström‐Öst et al. 2019) and cladocerans (Souza et al. 2007; Goswami et al. 2013). Using mesocosms, Zervoudaki et al. (2024) reported that LPX activity in the zooplankton community was high during a heatwave and increased further during post‐heatwave. Salinity also influenced LPX, probably due to evaporation. The catalase (CAT) activity was higher during post‐heatwave, while the GST activity showed no significant response to heatwave or salinity. However, previous studies have not investigated how changing salinity and temperature influence the zooplankton community's oxidative stress and antioxidant defence system via biomarker analyses in the natural marine environment.

The current work aims to assess eco‐physiological responses of the mesozooplankton community to environmental temperature, salinity, oxygen and chlorophyll *a* and how they influence the oxidative damage and the plasticity of antioxidants along a coastal zone in the Baltic Sea. In this study, we focus on plankton communities rather than species, aiming to identify biomarker responses that provide a more comprehensive picture of ecosystem health. Biomarkers were used as proxies to study how mesozooplankton respond to the environment. We hypothesised that oxidative stress would occur during warming, changing salinity, chlorophyll *a*, and hypoxia and that these responses would correspond to the temporal and spatial scales examined. Finally, we hypothesised that oxidative stress and the antioxidants would respond differently in different mesozooplankton communities, depending on which groups are the most abundant in the community (Souza et al. 2007; Vuori et al. 2015; Engström‐Öst et al. 2019).

## 2 Material and Methods

### 2.1 Field Sampling

**FIGURE 1:**
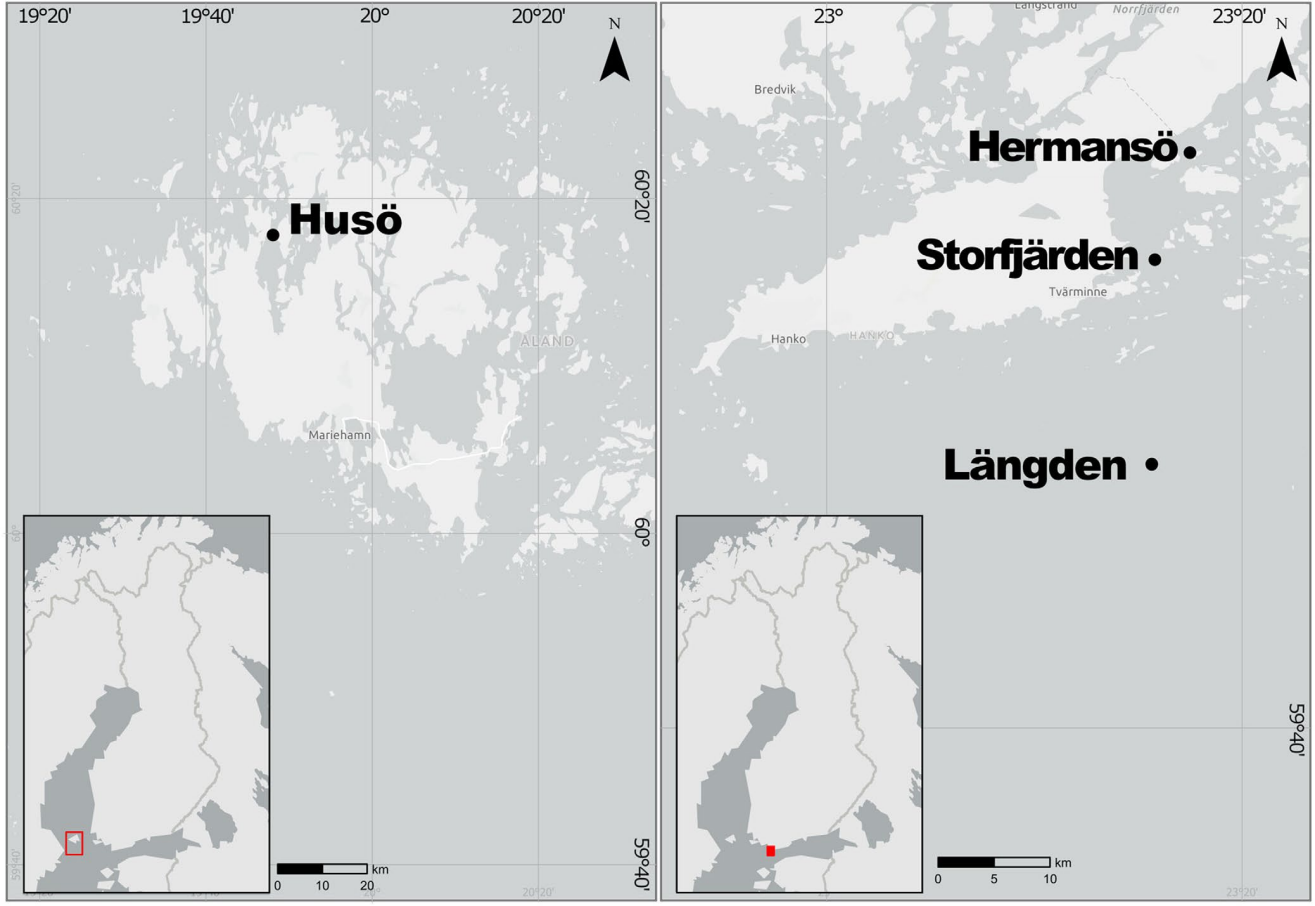
(i) The location of our sampling sites in the map of Europe (left insert), (ii) the sampling site in the map of the southwest part of Finland (right insert), (iii) the sampling site close to Husö Biological station, Åland Islands (left), (iv) the three sites close to Tvärminne Zoological Station (right). Basemap source: ESRI, 206 EPSG 4326 ‐ Noel Aurelie.

We collected mesozooplankton samples from four locations that vary in environmental conditions (chlorophyll a, temperature, salinity and oxygen; Figure 1; Cederberg 2021; von Weissenberg, Mottola, et al. 2022): three stations near Tvärminne Zoological Station in the south‐western Gulf of Finland, and one site close to Husö Biological Station, Åland Islands. The locations along the coastal zone were selected based on their geographic position, depth and the variability of the environmental conditions. From the areas near Tvärminne, Storfjärden is pelagic with a maximum depth of 35 m, Hermansö is a shallow and narrow area with a maximum depth of 15 m and Längden is an offshore site with a maximum depth of 55 m. Husö is a semi‐deep area in the inner archipelago of Åland Islands with a maximum depth of 25 m, which is known for seasonal hypoxia (Cederberg 2021). The samples were collected from July to September 2023 (Table 1).

**TABLE 1:** Summary of field sampling. The coordinates of the locations where the samples were collected and the dates of each sampling are noted. The maximum depth is represented for each location. The median temperature, salinity, dissolved oxygen, and chlorophyll *a* are noted for each location and date.

Location	Coordinates	Date	Depth (m)	Temperature (°C)	Salinity	Dissolved oxygen (mg/L)	Chlorophyll *a* (μg/L)
Storfjärden	59°51.194′ N, 23°15.481′ E	10/07/2023	35	7.47	6.31	10.57	4.01
		19/07/2023	35	8.85	6.26	10.09	2.75
		25/07/2023	35	7.88	6.28	9.64	1.83
		14/08/2023	35	17.52	6.34	7.9	7.92
Hermansö	59°53.904′ N, 23°17.490′ E	11/07/2023	15	10.82	6.2	9.82	2.54
		20/07/2023	15	11.79	6.17	10.1	3.09
		26/07/2023	15	10.22	6.21	9.6	2.08
Husö	60°17.817′ N, 19°48.065′ E	01/08/2023	25	18.6	5.49	5.43	7.54
		02/08/2023	25	19.15	5.48	6.21	8.58
		03/08/2023	25	19	5.48	6.53	10.19
Längden	59°46.385′ N, 23°15.652′ E	28/08/2023	55	18.13	6.32	7.66	5.33
		11/09/2023	55	12.93	6.54	7.27	3.35
		18/09/2023	55	12.06	6.56	7.81	2.87

Temperature and salinity were measured with CastAway‐CTD (Xylem Water Solutions, USA) from the bottom to the surface. Dissolved oxygen (DO) was measured with a YSI ProOdo meter (Xylem Water Solutions, USA), from every 5 m (von Weissenberg, Mottola, et al. 2022). In Husö the YSI ProODo meter was used to measure salinity and temperature. A Secchi disk (Avantor, USA) was used to measure water transparency. We collected samples for chlorophyll *a* with a 2.8 L Limnos water sampler (Hydro‐bios, Germany), from different depths depending on transparency measurements (Table 2, Kauppila and Kallio 2018), following the monitoring guidelines set by EU Marine Strategy Framework Directive (2008). Chlorophyll *a* concentration was measured by filtering a known volume of water on glassfibre filters (Whatman GF/C, Cytiva, USA), which were frozen in −20°C until analysis. Ethanol (10 mL, 96%) was added for the 24 h sample extraction, and the absorbance of the samples was measured with a spectrophotometer (Varian Cary Eclipse Fluorescence Spectrophotometer, Varian Optical Spectroscopy Instruments, Australia) (Arvola 1981; Engström‐Öst et al. 2015).

**TABLE 2:** The transparency defines from which depths the chlorophyll *a* samples were collected. We used a Secchi disk to determine transparency.

Secchi	< 1 m	1.1–2 m	2.1–3 m	3.1–4 m	> 4.1 m
Depth (m)	0, 0.5, 1, 1.5, 2	0, 1, 2, 3, 4	0, 2, 4, 6	0, 2, 4, 6, 8	0, 2, 4, 6, 8, 10

We used a zooplankton net (Hydro‐bios, Germany) with cod‐end, with a mesh size of 200 μm and diameter of 0.5 m to collect six mesozooplankton samples per sampling day from the bottom to the surface of each location. This collection method targeted mostly copepods, cladocerans, some rotifers, and larger larvae, while microzooplankton (smaller rotifers, nauplii, ciliates, and protozoans) passed through the net due to their smaller size relative to the mesh. The samples were kept in ~10 L of seawater from below the thermocline. The animals were sieved to remove water, using a 200 μm mesh size sieve, and stored, in triplicate per sampling time, for biomarker analyses (lipid peroxidation LPX, glutathione‐s‐transferase GST, and catalase CAT). The collection of the community lasted < 4 h until snap‐frozen in liquid nitrogen and stored in a −80°C freezer. We stored an additional mesozooplankton sample per location for community analysis, taken from 10 L of seawater. The sample was preserved in buffered formaldehyde seawater solution, with a final concentration of 4%. The identification of the community composition (> 200 μm) was conducted under a stereoscope (Nikon SMZ800, Japan). The magnification varied during the identification of the community, for which the entire sample was analysed, and all species found were identified. The zooplankton sampling took place in different areas from July to September; each sampling area was sampled 3–4 times within this period.

**FIGURE 2:**
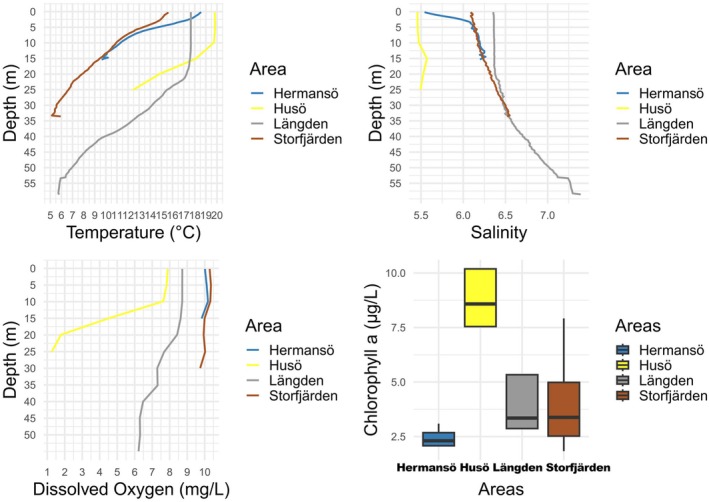
Environmental data measurements in the sampling sites. (i) Temperature (°C), (ii) salinity, (iii) concentration of dissolved oxygen (mg/L), (iv) chlorophyll *a* (μg/L). At the *y* axis, of a, b and c is the depth (m). The graphs show the averages of all sampling days of each site separately.

Based on the environmental data, we calculated the averages at each depth and site sampled, showing the depth profiles of data from bottom to surface (Figure 2).

### 2.2 Biomarker Analyses

The frozen zooplankton samples were crushed and subsamples were weighed for subsequent analysis. The samples were kept frozen in liquid nitrogen. Each sample was homogenised in 100 mM K‐phosphate buffer 5 μL/1 mg of the samples, with the Bullet Blender tissue homogeniser (Next Advance Inc., USA) at 4°C for 8 min. The samples were split for 5 different analyses using raw homogenate or centrifuged homogenate supernatant (4°C, 10,000 *g* for 15 min). The raw homogenate subsamples were used for lipid peroxidation (LPX) and bicinchoninic acid (BCA raw) protein assay and centrifuged subsamples were used for BCA, glutathione‐s‐transferase (GST) and catalase (CAT) assays. All subsamples were immediately frozen in liquid nitrogen and stored in −80°C until further analyses.

The protein level (BCA) of the homogenised samples was determined by using the BCA protein assay kit (Pierce, Thermo Scientific, USA). The BCA spectrophotometric measurements were performed at 570 nm. The protein measurements were used to normalise the LPX, GST, and CAT per protein concentration.

Lipid peroxidation assay (LPX) was determined using the ferrous oxidation‐xylenol orange method (Vuori and Kanerva 2018a). Briefly, the homogenate was diluted in methanol and incubated for 10 min, then the sample was centrifuged at 3000 *g* for 5 min. The supernatants were collected and methanol was added to reach a final volume. The samples were incubated for 30 min at room temperature. Afterwards, the reaction mix was added and the samples were incubated for 2 h at room temperature in the dark. The reaction mix consisted of 1 part of 2.5 mM ammonium iron (II) sulfate in 0.25 M H_2_SO_4_ and 9 parts of 0.111 mM xylenol orange in methanol. Finally, the absorbance was measured at 570 nm.

Glutathione‐s‐transferase assay (GST) was determined according to the protocol of Habig et al. (1974), with modifications as described in Vuori et al. (2015). Briefly, 1 μL of the sample was mixed with 49 μL of the reaction mix, that consisted of 2 mM reduced glutathione and 1 mM 1‐Chloro‐2,4‐dinitrobenzene diluted in Dulbecco's Phosphate Buffered Saline, and the reaction concentration with sample being 1.98 and 0.98 mM respectively. The plate was shaken for 5 s and the absorbance was read at 340 nm for 6–13 min.

Catalase assay (CAT) was determined using the protocol of Vuori and Kanerva (2018b). The samples were diluted to 0.6 mg/mL protein with the 50 mM KF‐buffer. The reaction was started by adding 50 mM H_2_O_2_ and allowed to proceed for approximately 4 min, then reaction terminated using 15 mM NaN_3_ in MQ‐H_2_O. Then, the samples were incubated in the dark for 15 min. Finally, they were measured at 520 nm.

Spectrophotometric measurements were performed with a Wallac EnVision 2103 Multilabel Reader (Perkin Elmer, Turku, Finland). All the samples, standards, and blanks were analysed in triplicate. Plate controls were used for the GST and CAT to analyse the variability between the plates (< 20%). If the mean coefficient variation percentage (CV%) was over 10 for each triplicate, the analyses were discarded and redone.

### 2.3 Statistical Analyses

Using depth profile data, we calculated the medians for statistical comparisons between locations. Furthermore, as the depth profiles were significant between locations at the surface, we also compared the environmental data between locations at 10 m depth, as many zooplankton species perform diel vertical migration, during which surface‐layer conditions can influence their physiological response (Glippa et al. 2018). We defined the surface layer as the upper 0–10 m of the water column. For plotting the graphs of the mesozooplankton community, we used averages from July to September for all locations. However, these averages were not used in the statistical analyses, but we always used the data from the corresponding sampling day. Linear Mixed Models (LMM) were used to analyse how median environmental data (temperature, salinity, dissolved oxygen mg/L, chlorophyll *a*) at spatial and temporal scales influenced the biomarkers (LPX, CAT and GST). The normality (Shapiro–Wilk test) and the homoscedasticity (Levene's test) were checked prior to LMM and Kruskal–Wallis test. In the LMM, we used data from 10 m depth to define the surface layer, and the median, to study the effects of the whole water column. The used packages were *lme4* (Bates et al. 2015) and *Matrix* (Bates et al. 2024) for the LMM. Location was used as a random effect and the dates were used as nested random effects within locations in the LMM. Before each LMM, the Akaike information criterion (AIC) was calculated and the lowest value was used for the LMM. The impact of sampling days on biomarkers was tested for each location separately with the Kruskal–Wallis test (*dunn. test*, Dinno 2024). Differences in antioxidants, lipid peroxidation and protein between locations were also tested with the Kruskal–Wallis test, and the Bonferroni method was used for pairwise comparisons.

PCA and RDA were used to analyse the relationships among environmental data, biomarkers and mesozooplankton species. PCA was used to identify patterns among biomarkers, environmental conditions, and species, while RDA was used to examine how environmental conditions influenced the relationships between biomarkers and species. The packages that were used for principal component analysis (PCA) were the *corrr* (Kuhn et al. 2022), *ggcorrplot* (Kassambara 2023), *FactorMiner* (Le et al. 2008) and *factoextra* (Kassambara and Mundt 2020). The *vegan* package was used for redundancy analysis (RDA) (Oksanen et al. 2025). The correlations were checked before each PCA. At the end, we calculated the ratio of cladocerans and copepods and performed a regression analysis for the relationship between this ratio and the biomarkers. R studio (version: 2024.12.0 + 467; R Core Team 2022) was used for the statistical analyses and Microsoft 365—Excel was used for the zooplankton community graph. Package *ggplot2* was used for other graphs (Wickham 2016).

## 3 Results

### 3.1 Hydrography and Zooplankton Community

The waters of Husö were warmer in comparison with the other areas; also, salinity was slightly lower and the bottom water layer was almost anoxic, the average for all days was 1.25 ± 0.51 mg/L (average ± SD) (Figure 2). The average SST at Längden was ~18°C and at 55 m the water temperature was 6.38°C ± 0.79°C; the thermocline was at 35 m depth. Längden had a higher salinity than the other areas and the oxygen concentration varied between 6 and 9 mg/L throughout the water column with few changes between days. Storfjärden and Hermansö had almost the same temperature, salinity, and DO, but the surface at Hermansö tended to have higher temperature and lower salinity. The thermocline at Storfjärden was consistently observed at shallow depths, ranging from 3.8 to 5 m across all sampling dates. In contrast, Längden showed notable variability in thermocline depth, recorded at 34 m on 28 August, at 18 m on 11 September, and at 22 m on 18 September. At Husö, the thermocline was detected at 10 m depth. No clear thermocline was present at the shallow site, Hermansö. All environmental data are shown in Table 1.

Husö had a higher concentration of chlorophyll *a* in comparison with the other locations (Figure 2, Table 1). On the other hand, chlorophyll *a* at Hermansö was lower in comparison with other areas. Among all sampling days at Hermansö, chlorophyll *a* was the highest at the end of July. Chlorophyll *a* in Längden dropped in September. Finally, the highest concentration of chlorophyll *a* at Storfjärden was in August.

**FIGURE 3:**
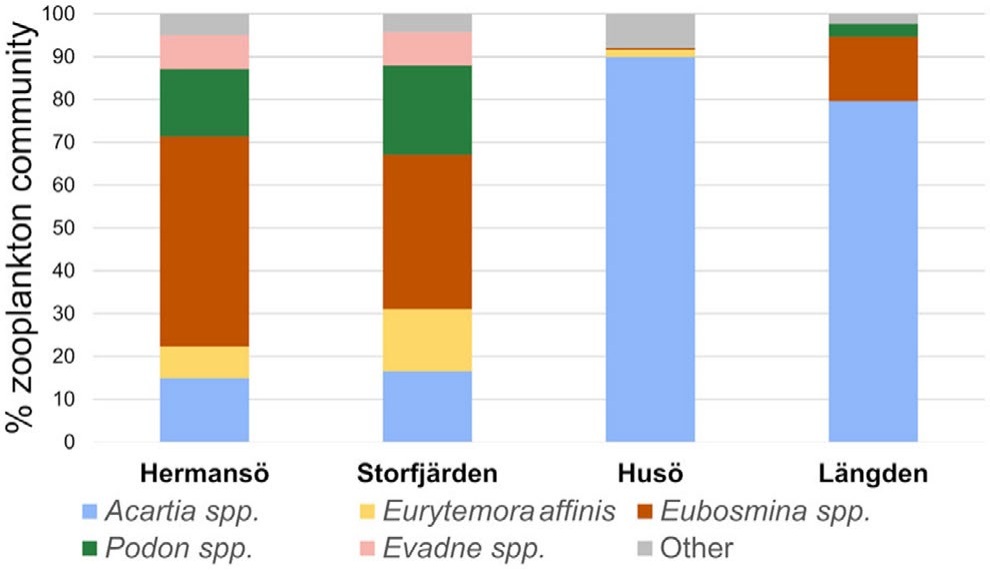
The percentage (%) of the average abundance of zooplankton species across sampling days at each sampling site. The most abundant species are Acartia spp., *Eurytemora affinis* , Eubosmina spp., Podon spp., Evadne spp. and all the other species, that were not as abundant in any of the sampling sites. The columns represent the average abundance of the whole community for all sampling days. Other species, detected at the sampling locations, were < 10%. In Storfjärden and Hermansö, we detected Balanus, Keratella, and polychaetes. Gastropods were also found in Storfjärden. In Husö, crab larvae were observed, and bryozoa larvae were found in Längden.

The mesozooplankton community consisted of *Acartia* spp., *Eubosmina* spp., *Eurytemora affinis* , *Evadne* spp. and *Podon* spp. (Figure 3). Storfjärden and Hermansö shared the same dominant species, which mainly consisted of cladocerans (Hermansö 73% and Storfjärden 65%). Husö and Längden stations were dominated by copepods, *Acartia* spp. (Husö 92% and Längden 80%).

### 3.2 Spatio‐Temporal Changes in Biomarkers

**FIGURE 4:**
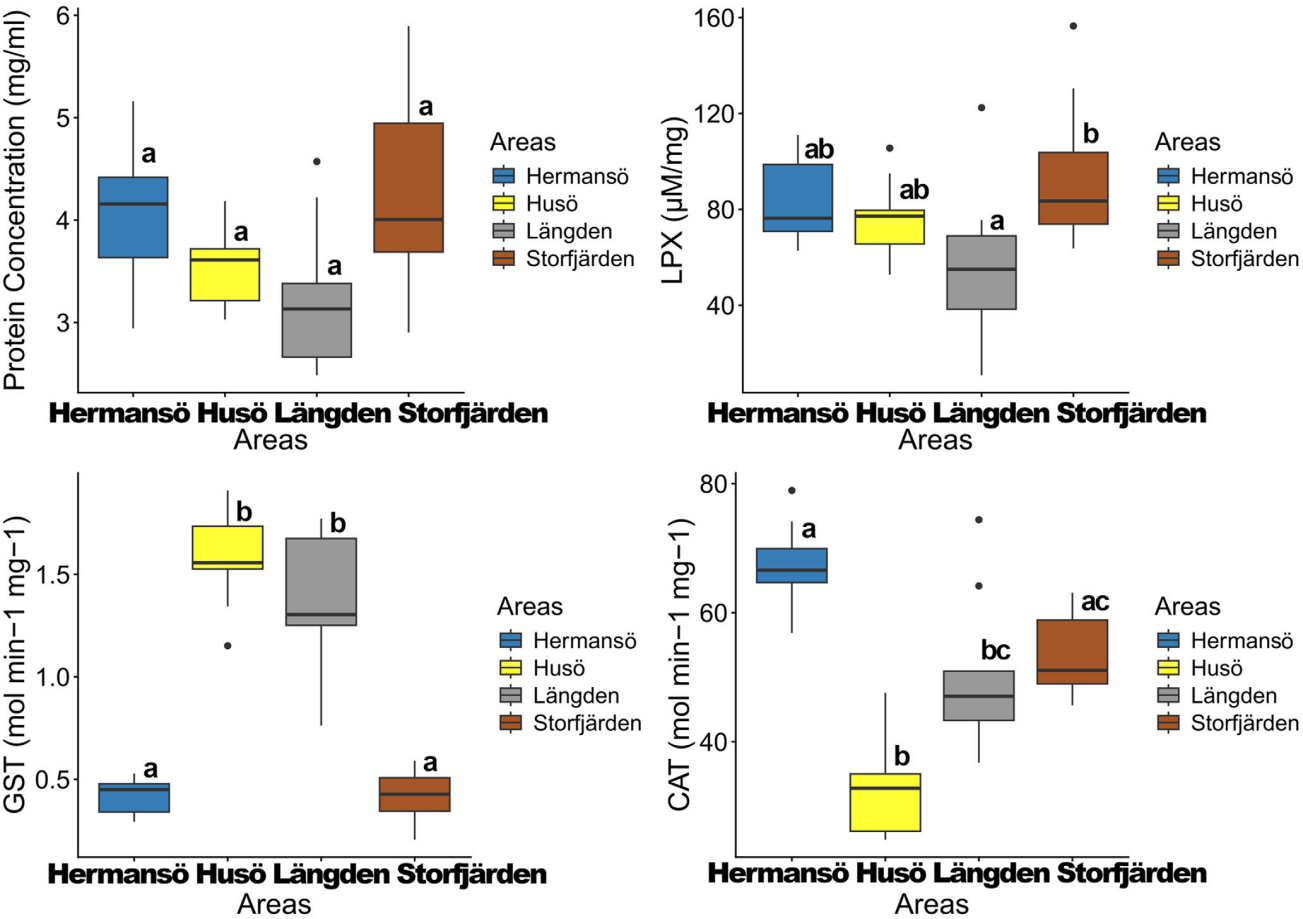
(i) CAT—catalase activity, (ii) GST—Glutathione‐s‐transferase activity, (iii) LPX—lipid peroxidation, indicated by concentration of ferrous oxidation—xylenol orange, (iv) protein concentration, these are the averages from the sampling dates per site. i–iii are shown per mg of protein in the sample. We used the letters a, b, c to show the significant differences (*p*‐value < 0.05), when two letters are together or the same, there are no significant differences between the areas.

The GST activity varied between sites (Kruskal–Wallis test *χ* ^2^ = 30.43, df = 3, *p* < 0.001); post hoc comparisons showed that the GST activity of the total community was significantly higher in Husö and Längden, in comparison to Hermansö and Storfjärden (Bonferroni, *p* < 0.05; Figure 4). The CAT activity of the total community was significantly different between all areas (Kruskal–Wallis test *χ* ^2^ = 28.95, df = 3, *p* < 0.001, Bonferroni *p* < 0.05), except between Storfjärden and Längden (Figure 4). The highest activity in CAT was at Hermansö (66.91 ± 5.85 mol min^−1^ mg^−1^) and the lowest in Husö (32.6 ± 7.52 mol min^−1^ mg^−1^). On the other hand, the LPX of the community was significantly higher in Storfjärden compared to Längden (Kruskal–Wallis test *χ* ^2^ = 8.73, df = 3, *p* = 0.03, Bonferroni *p* < 0.05; Figure 4). The protein concentration of the community in all areas was almost at the same level (*p* > 0.05), despite the different environmental conditions. There was no significant effect found by sampling day on CAT or LPX (Kruskal–Wallis test, *p* > 0.05). However, GST showed a significant difference between the first and last sampling day in Hermansö (Kruskal–Wallis test, *χ* ^2^ = 7.60, df = 2, *p* = 0.022).

### 3.3 Biomarkers and Environmental Conditions

**FIGURE 5:**
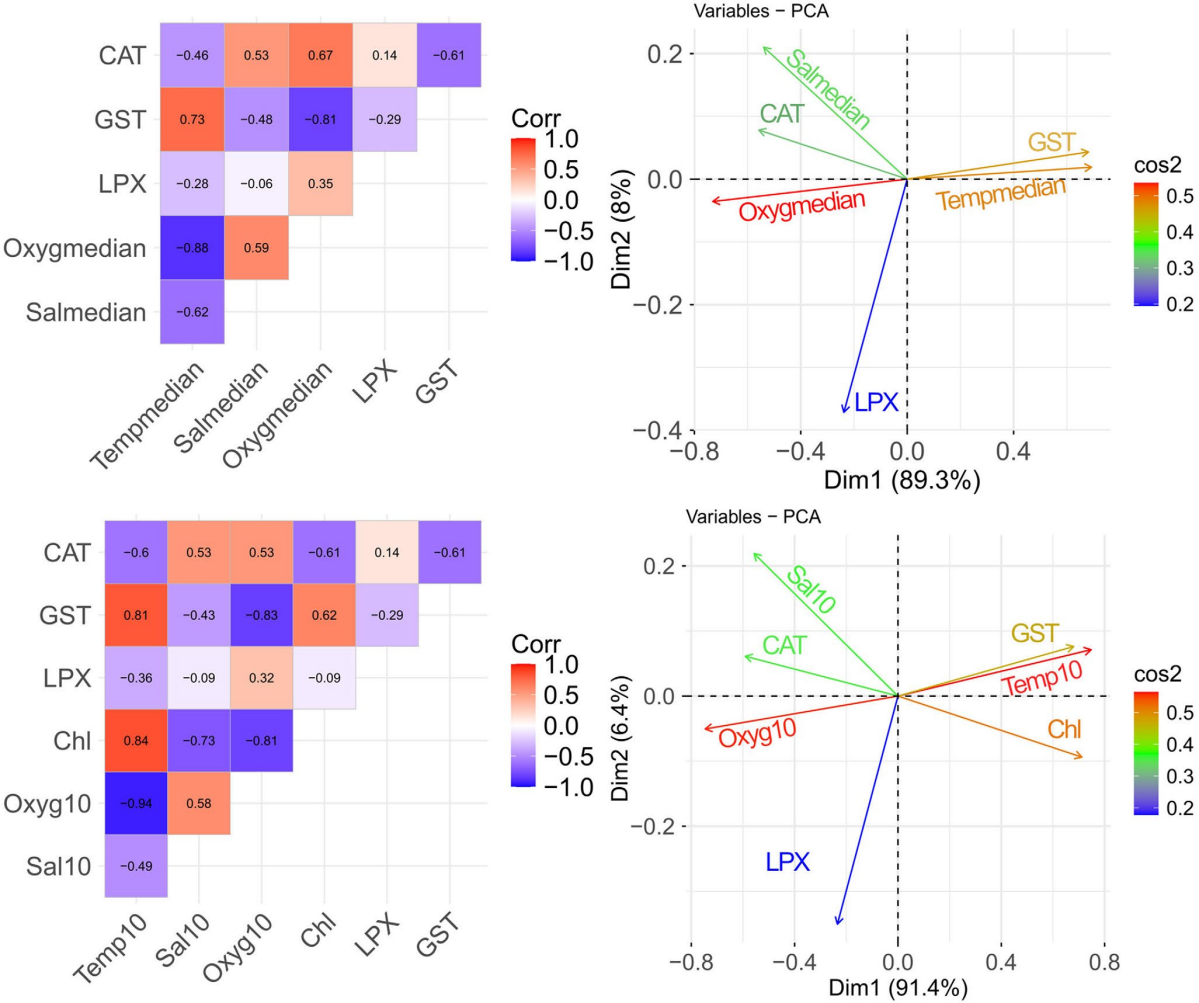
(i) Correlations and (ii) PCA between median temperature (Tempmedian), median salinity (Salmedian) and median dissolved oxygen (Oxygmedian) and Catalase activity (CAT), Glutathione‐s‐transferase activity (GST) and (LPX). (iii) correlations and (iv) PCA between temperature at 10 m (Temp10), salinity at 10 m (Sal10) and dissolved oxygen at 10 m (Oxyg10), chlorophyll *a* (Chl) and Catalase activity (CAT), Glutathione‐s‐transferase activity (GST) and (LPX). (iii) Correlations. The blue colour in the correlations shows a positive correlation and the red shows a negative one. The colours in PCAs show which one attributes more in PCA, the red attributes the most, the blue the least and the green is mid.

The variables that contributed to both PCA analyses were the temperature and DO (median and at 10 m) (Figure 5). The GST showed a negative correlation with median and at 10 m DO and a positive correlation with median and at 10 m temperature. On the other hand, CAT was positively correlated with median DO. In the PCA, CAT was negatively correlated with chlorophyll *a*. The LPX was not affected by the environmental variables while using the median and at 10 m depth in the PCA analysis. The RDA plot shows that GST strongly correlated with temperature (Figure S1). CAT was associated with low salinity and lower oxygen values, whereas LPX was associated with dissolved oxygen (Figure S1).

Based on LMM analyses, temperature at 10 m was a significant predictor of LPX. Also, median dissolved oxygen was a marginally significant predictor for GST. Median temperature and dissolved oxygen were marginally statistically significant predictors of CAT activity. Dissolved oxygen at 10 m was a significant predictor of CAT activity. Salinity and chlorophyll *a* were not significant predictors for any antioxidant or lipid peroxidation (Table 3).

**TABLE 3:** Linear mixed model's results at median and 0–10 m depth.

		Dependent variables	Estimate	Std. Error	df	*t*	*p*	AIC
Median	LPX	(Intercept)	192.66	111.53	7.56	1.73	0.125	
		Temperature	−0.20	1.92	37.93	−0.11	0.916	394.54
		Salinity	−30.67	14.91	3.27	−2.06	0.124	392.18
		Dissolved oxygen	9.14	5.17	12.07	1.77	0.103	390.69
	CAT	(Intercept)	−121.20	86.86	1.70	−1.39	0.317	
		Temperature	2.11	1.02	6.96	2.06	0.079	338.96
		Salinity	14.11	12.75	1.55	1.11	0.411	335.63
		Dissolved oxygen	6.887	3.37	7.83	2.04	0.076	316.4
	GST	(Intercept)	3.28	4.43	8.28	0.74	0.479	
		Temperature	0.03	0.02	9.24	1.63	0.137	44.91
		Salinity	−0.70	0.63	8.52	−1.11	0.297	46.81
		Dissolved oxygen	0.18	0.08	9.62	2.06	0.067	35.28
0–10 m	LPX	(Intercept)	319.19	97.12	3.00	3.29	0.046	
		Temperature	−4.40	1.43	4.09	−3.08	**0.036**	392.31
		Salinity	−28.31	13.94	2.66	−2.03	0.147	389.67
	CAT	(Intercept)	503.07	182.98	8.57	2.75	0.023	
		Temperature	−1.91	1.23	4.28	−1.55	0.192	330.26
		Salinity	−52.70	29.85	8.59	−1.77	0.113	327.2
		Dissolved oxygen	−11.50	4.21	4.23	−2.73	**0.049**	325.01
	GST	(Intercept)	6.88	5.67	6.54	1.66	0.267	
		Temperature	−0.11	0.06	6.67	−1.66	0.142	32.41
		Salinity	−0.70	0.92	6.18	−0.76	0.475	34.25
		Oxygen	−0.06	0.15	6.02	−0.37	0.726	31.04
		Chlorophyll *a*	0.11	0.07	6.83	1.65	0.144	30.88

*Note:* Catalase enzyme (CAT), glutathione‐s‐transferase enzyme (GST), and lipid peroxidation (LPX) were the dependent variables, and the continuous variables were the temperature, salinity, dissolved oxygen, and the chlorophyll *a*. Sampling areas were used as a random effect, and the dates were used as a nested random effect. The lowest AIC values were used to select the best model to run. Marginally significant results are *p* = 0.05. Significant *p*‐values are denoted *p* < 0.05.

### 3.4 Biomarkers and Zooplankton Community

**FIGURE 6:**
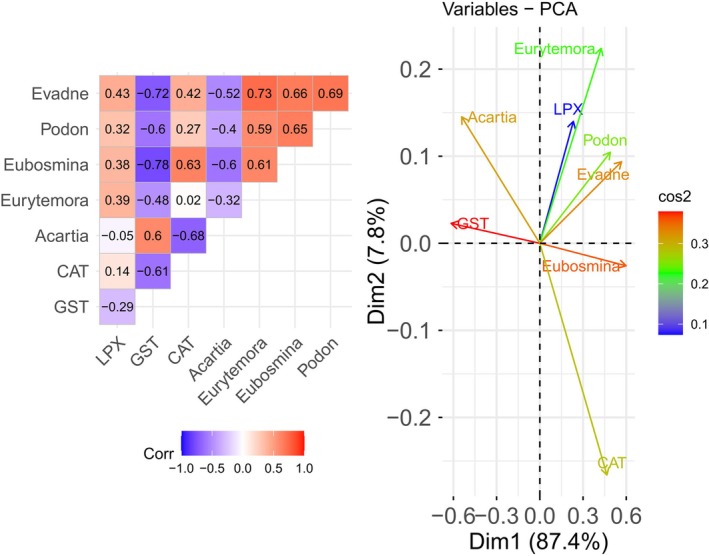
(i) Correlations and (ii) PCA between *Acartia* spp., *Eurytemora affinis* , *Eubosmina* spp., *Podon* spp., and *Evadne* spp. and Catalase activity (CAT), Glutathione‐s‐transferase activity (GST) and (LPX). (iii) Correlations. The blue colour in the correlations shows a positive correlation and the red shows a negative one. The colours in PCAs show which one attributes more in PCA, the red attributes the most, the blue the least and the green is mid.

The variables that contributed most strongly to the PCA were the GST and *Eubosmina* spp. GST was positively correlated with *Acartia* spp. and negatively correlated with the *Eubosmina* cladocera, whereas CAT showed the opposite pattern (Figure 6). LPX of the total community did not vary depending on the mesozooplankton composition. The RDA plot shows that GST strongly separates *Acartia* spp. from the other mesozooplankton, whereas CAT correlated with *Eubosmina* spp., and the remaining species exhibited weak responses to these biomarkers. LPX did not show a strong correlation with any species (Figure S2).

The regression analysis between the ratio of cladocerans/copepods and LPX showed a weak positive trend, although the relationship was not statistically significant, whereas the ratio of cladocerans/copepods and GST showed a strong negative relationship. On the other hand, the ratio of cladocerans/copepods and CAT showed a strong positive relationship (Table 4).

**TABLE 4:** The results of the regression analysis between the ratio of cladocerans/copepods and catalase enzyme (CAT), glutathione‐s‐transferase enzyme (GST), and lipid peroxidation (LPX).

	Estimate	Std. Error	*t*	*p*
Intercept	0.290	0.799	0.364	0.718
LPX	0.018	0.009	1.900	0.065
Intercept	3.721	0.298	12.498	**< 0.001**
GST	−2.272	0.286	−7.931	**< 0.001**
Intercept	−2.624	0.657	−3.996	**< 0.001**
CAT	0.083	0.012	6.884	**< 0.001**

*Note:* Significant *p*‐values are denoted *p* < 0.05.

## 4 Discussion

In the current work, we hypothesised that the antioxidant activity of mesozooplankton communities responds to different environmental conditions. The zooplankton community, DO and temperature were identified as the primary drivers affecting antioxidant responses. The community and DO variables were closely associated with both CAT and GST activities, whereas temperature additionally showed a significant contribution to GST and oxidative stress, measured here as lipid peroxidation (LPX) variation. LPX did not show any correlation with other environmental variables. In contrast to our hypothesis, lipid peroxidation remained low and stable, possibly because we did not observe any heatwave or significant changes in salinity during summer 2023. Finally, oxidative stress levels remained stable and mesozooplankton were able to keep them low.

During normal oxygen consumption, oxygen free radicals are produced, which can damage vital biological molecules such as nucleic acids, proteins, and lipids (Harman 1956; Hulbert et al. 2007). This damage contributes to the ageing process, and over time, oxygen free radicals accumulation ultimately leads to the death of the individual. GST protects cellular macromolecules from reactive electrons, which are included in the Phase II detoxification enzymes (Townsend and Tew 2003). In our work, GST activity differed significantly between stations and correlated positively with temperature and chlorophyll *a*, and negatively with DO, while salinity did not seem to have any effect on GST. Our results with temperature are in line with other studies since crustacean zooplankton, such as cladocerans and copepods, are shown to respond to temperature in terms of GST (Cailleaud et al. 2007; Wolinski et al. 2016; Glippa et al. 2018). The GST activity response rate in copepods is relatively fast, probably less than one hour, and can also vary over depths (Glippa et al. 2018). Previous work shows that GST was activated in both *Acartia* spp. and *Eurytemora affinis* during warming and salinity increase in the field and experiments (Cailleaud et al. 2007; von Weissenberg, Mottola, et al. 2022; von Weissenberg, Jansson, et al. 2022). Both shrimp ( *Litopenaeus vannamei* ) and fish ( *Acipenser naccarii* and *Paralichthys olivaceus* ) can adapt to changes in salinity due to the antioxidant defence, which demonstrates how the free radical processes are affected by seawater (Lushchak 2011 and references therein). This could be one reason why we did not find any correlation between GST and salinity, despite significant differences in salinity level of the sites.

D'Aiuto et al. (2022) reported that more severe hypoxia is expected to reduce reactive oxygen species (ROS), and more mild hypoxia or normoxia could increase ROS in cells. On the other hand, both hypoxia (< 2 mg/L) (NOAA 2025) and hyperoxia (> 10 mg/L) can cause oxidative stress (McArley et al. 2020). Our results about GST being negatively connected to DO are supported by D'Aiuto et al. (2022), because the GST activities in mesozooplankton in our study were high in Husö, likely because the animals were exposed to hypoxia in Husö, a site known for temporal oxygen deficiency. Also, corresponding results were observed, especially at Storfjärden, where the GST activity decreased when the DO was elevated. According to Vuori et al. (2015), DO influenced GST activity in *Limnocalanus macrurus* ; when oxygen was high, the GST increased, and when the environment was less saline and DO was higher, the GST activities increased further. This discrepancy indicates that the relationship between DO and GST activity may be context dependent, with additional environmental variables potentially modifying GST responses. Indeed, in our study, GST also correlated strongly with chlorophyll *a* across all sites. During some sampling days, we observed a thick layer of the filamentous cyanobacterium *Aphanizomenon flos‐aquae* in Husö. Gorokhova and El‐Shehawy (2022) reported that the concentration of cyanobacteria in mixed foods can activate the GST in aquatic crustaceans.

Our findings that GST is correlated with Acartia spp. and that GST has a negative connection with the cladocerans/copepods ratio are supported by Vuori et al. (2015) and Engström‐Öst et al. (2019), who showed that elevated levels of glutathione metabolism, such as GST, can be induced by hydrographic conditions in the copepod *Acartia* spp. Due to their diel vertical migration, copepods encounter wide environmental ranges daily (Almén et al. 2014), possibly increasing their resilience towards a rapidly changing environment, and in combination with the plasticity of the antioxidants, it allows copepods to adapt and survive. Cladocerans, on the other hand, migrate little and dwell mostly in the upper waters (Burris 1980). Therefore, the community composition and the cladoceran/copepod ratio can partly explain our results, such as why GST increased in low DO concentrations in Husö, when the community mainly consisted of *Acartia* spp. GST was lower in cladoceran‐dominated communities, shown by the cladoceran/copepod ratio. Another factor that may affect the cladoceran/copepod ratio at the different sites is the effect of estuarine outflow; especially Hermansö and Storfjärden are under the potential influence of River Svartån, which water passes the sampling sites on its way offshore (Katajisto et al. 1998).

Catalase (CAT) is an antioxidant enzyme that converts hydrogen peroxide H_2_O_2_ to water and oxygen (Switala and Loewen 2002). In our results, CAT was significantly related to salinity and dissolved oxygen as well as chlorophyll *a*, and it also depended on the composition of the community. The salinity results are supported by Glippa et al. (2018) who found that CAT activity in *Acartia* spp. increased as a response to lower salinity in the Baltic Sea. The significant relationship between CAT and DO may be due to the fact that there is a wide range of oxygen levels in our sampling sites: from hyperoxia at the surface layer to anoxia at the bottom water layer. Our results are, however, in contrast with Borgeraas and Hessen (2000) who found no relationship between DO and activity of CAT in cladoceran *Daphnia magna*; thus, more research is needed. Another factor having an effect could be cyanobacteria blooms. Our results showed a negative correlation between CAT and chlorophyll *a*. Pinto et al. (2024) observed that the CAT activity of freshwater *D. magna* juveniles decreased due to exposure to cyanobacterial toxin; the authors suggested that this was followed by an increase in GST activity. Similarly, the CAT activity of crustaceans and rotifers can respond to cyanobacteria blooms (Ferrão‐Filho et al. 2017; Liang et al. 2021; Gorokhova and El‐Shehawy 2022). We also found a pattern of higher CAT activity during higher cladoceran/copepod ratios. Souza et al. (2007) and Goswami et al. (2013) observed that the CAT activity was lower in copepods in comparison with cladocerans, supporting our results. Taken together, the antioxidant responses (both GST and CAT) seem to be affected by multiple factors, but when it comes to community‐level responses, the composition of the community and the environmental oxygen status probably play the biggest roles.

Oxidative stress can lead to sublethal responses, such as changes in fitness, growth and survival (von Weissenberg, Jansson, et al. 2022). LPX is oxidative degradation of cellular lipids, resulting in cell damage (Mylonas and Kouretas 1999). In our study, LPX activity remained stable in all sampling sites and did not correlate with any mesozooplankton species, but we found a significant relationship between LPX and the temperature at 10 m depth. The results obtained by von Weissenberg, Mottola, et al. (2022) and von Weissenberg, Jansson, et al. (2022) support the importance of temperature at 10 m in our results, suggesting the temperature is close to optimal at that depth. They further report that LPX in *Acartia* spp. seems to be low in ambient temperature (9°C), which was used in the experiment. However, LPX increased in elevated temperature and kept rising during a prolonged heatwave. Vuori et al. (2015) observed low LPX in the glacial relict *Limnocalanus macrurus* during high DO and low temperature in the Bothnian Sea. Therefore, in our study, despite the antioxidants fluctuating between the different locations and under different environmental conditions, lipid peroxidation remained stable, likely because the antioxidant production was activated and functioned well. The stability of lipid peroxidation in the field could be due to the fact that no heatwave was detected in the study area during sampling, as lipid peroxidation is known to be a sensitive measure, especially during warming.

In general, it needs to be noted that sampling was conducted between July and September, which can explain the differences in environmental conditions between the locations. These environmental differences might affect the antioxidative stress enzymes and oxidative marker differences over time and between studied sites. However, overtime comparisons revealed that time, by itself, had little effect on CAT and LPX, although GST activity seemed to be affected by sampling time, but only in Hermansö. We assume that the change in zooplankton composition over time in Hermansö is the reason why GST activity varied there. The number of Acartia, which have high GST activity as compared to other copepods, in Hermansö increased over time; on the first sampling day there were 13 *Acartia* spp./L, while on the last sampling day there were 22 *Acartia* spp./L.

Another factor that might explain the differences noted between stations could be their depth because the community composition could differ depending on their depth. Copepods are sensitive to predation by fish and migrate vertically in the water column to be in the deeps during the day, and move towards the surface to feed during the night (Almén et al. 2014). The cladoceran species migrate considerably less than the copepods; *Evadne* sp. migrate to some extent, whereas *Bosmina* sp. and *Podon* sp. migrate little (Burris 1980). The cladoceran/copepod ratio is likely also affected by the site; in Husö, based on Åland Islands and that is a marine site, copepods were more abundant than cladocerans in the current work. Instead, the Tvärminne area is affected by the River Svartån and cladocerans were more abundant. Finally, Glippa et al. (2018) have previously shown that biomarker responses may vary between depths. They showed that glutathione antioxidants varied between depths, perhaps due to temperature, and also lipid peroxidation was higher in mid‐depths, perhaps as a response to low food quality.

One more point of consideration is how environmental conditions at the surface are related to community responses that represent the whole water column. We were considering the surface also as an important factor since many zooplankton species perform diel vertical migration, during which surface‐layer conditions can influence their physiological responses (Glippa et al. 2018). Since factors such as surface temperature and chlorophyll *a* concentration may affect the measured biomarker responses, we chose to analyse the environmental variables from the upper water layer (10 m depth) in relation to the biomarkers (von Weissenberg, Mottola, et al. 2022). Yet, these conditions might not affect the physiology of those zooplankton that migrate only little. Nevertheless, we found that in general both surface and median environmental conditions were both related to biomarkers. In future studies, it would be beneficial to include more sites with more variable environments, and it would be beneficial to obtain a clearer picture of which environmental variables are most essential. Further experimental studies are also needed where copepods and cladocerans are directly compared to each other. It would be also important to follow how the community structure changes over time in each station and how those are related to changes in environmental conditions. In the future microzooplankton/seston also need to be investigated as they are the main food for many mesozooplankton.

## 5 Conclusions

Our results suggest that the antioxidant defence seems plastic, and this plasticity varies depending on species composition and environmental conditions. This plasticity enabled the mesozooplankton communities to maintain low oxidative stress levels across fluctuating environmental conditions and community compositions. Main drivers in the current study were temperature, oxygen and zooplankton community. Our study focuses on antioxidants in mesozooplankton responding to different environmental conditions, with particular emphasis on responses at the level of the entire mesozooplankton community. Zooplankton, as part of the lower trophic levels, are essential grazers of the primary producers. If zooplankton mortality increases, fish recruitment may suffer. The antioxidants and oxidative stress measure health and wellbeing, and potential long‐term stress in organisms. Lipid peroxidation is a sensitive measure, but it is affected especially by the temperature. The stability of lipid peroxidation in the field could be due to the fact that heatwaves were not detected in the study area during the field season. Future work should focus on the responses of different biomarkers during advancing environmental change.

## Author Contributions

**Andriana Koutsandrea:** conceptualization (equal), data curation (equal), formal analysis (equal), investigation (equal), methodology (equal), visualization (equal), writing – original draft (lead), writing – review and editing (equal). **Tytti‐Maria Uurasmaa:** formal analysis (supporting), methodology (supporting), writing – review and editing (equal). **Katja Anttila:** formal analysis (supporting), methodology (supporting), resources (equal), writing – review and editing (equal). **Jonna Engström‐Öst:** conceptualization (equal), funding acquisition (equal), investigation (equal), methodology (equal), project administration (equal), resources (equal), supervision (equal), writing – review and editing (equal).

## Funding

This work was supported by Svenska Kulturfonden; Waldemar von Frenckells Stiftelse; Biotieteiden ja Ympäristön Tutkimuksen Toimikunta, nr. 361936.

## Ethics Statement

The zooplankton communities that were collected for this study are not endangered or protected species and therefore, no permit was needed for sampling.

## Conflicts of Interest

The authors declare no conflicts of interest.

## Supporting information


**Figure S1:** RDA biplot showing the relationships between environmental variables—median and 10 m temperature (Tmed, T10), median and 10 m salinity (Smed, S10), median and 10 m dissolved oxygen (Omed, O10) and chlorophyll a (Chl) and biomarker responses, including Catalase activity (CAT), Glutathione‐S‐transferase activity (GST), and lipid peroxidation (LPX). Blue arrows represent biomarker vectors, and red labels represent environmental variables. Axis 1 (RDA1) explains 50.4% of the variance, and Axis 2 (RDA2) explains 3.8%.
**Figure S2:** RDA biplot showing the relationships between *Acartia* spp., *Eurytemora affinis* , *Eubosmina* spp., *Podon* spp., and *Evadne* spp. and biomarker responses, including Catalase activity (CAT), Glutathione‐S‐transferase activity (GST), and lipid peroxidation (LPX). Blue arrows represent biomarker vectors, and red labels represent the species variables. Axis 1 (RDA1) explains 68.1% of the variance, and Axis 2 (RDA2) explains 5%.

## Data Availability

The datasets in the current work will be submitted to an open data repository upon acceptance. The raw data are uploaded as a Supporting Information for review.
