# Supplementary material for: The Plasticity of the Antioxidant Defence System of Coastal Zooplankton Communities
Source: Ecol Evol. 2026 Jun 28;16(7):e73919. doi: 10.1002/ece3.73919 (PMC13311195; doi:10.1002/ece3.73919)
Supplement: Supplementary file 1 — Figure S1: RDA biplot showing the relationships between environmental variables—median and 10 m temperature (Tmed, T10), median and 10 m salinity (Smed, S10), median and 10 m dissolved oxygen (Omed, O10) and chlorophyll a (Chl) and biomarker responses, including Catalase activity (CAT), Glutathione‐S‐transferase activity (GST), and lipid peroxidation (LPX). Blue arrows represent biomarker vectors, and red labels represent environmental variables. Axis 1 (RDA1) explains 50.4% of the variance, and Axis 2 (RDA2) explains 3.8%. Figure S2: RDA biplot showing the relationships between Acartia spp., Eurytemora affinis , Eubosmina spp., Podon spp., and Evadne spp. and biomarker responses, including Catalase activity (CAT), Glutathione‐S‐transferase activity (GST), and lipid peroxidation (LPX). Blue arrows represent biomarker vectors, and red labels represent the species variables. Axis 1 (RDA1) explains 68.1% of the variance, and Axis 2 (RDA2) explains 5%. [file ECE3-16-e73919-s001.pdf]

Supplementary materials

The plasticity of the antioxidant defence system of coastal zooplankton communities

Andriana Koutsandrea[1,2], Tytti-Maria Uurasmaa[3], Katja Anttila[3], Jonna Engström-Öst[2,4]

[1]Åbo Akademi University, Henriksgatan 2, FIN-20500 Åbo, Finland

[2]Novia University of Applied Sciences, Raseborgsvägen 11, FIN-10600 Ekenäs, Finland

[3]Department of Biology, University of Turku, Vesilinnantie 2, FIN-20014 Turku, Finland

[4]Tvärminne Zoological Station, J.A. Palméns väg 160, FIN-10900 Hangö, Finland

E-mail of corresponding author: Andriana Koutsandrea, andriana.koutsandrea@abo.fi

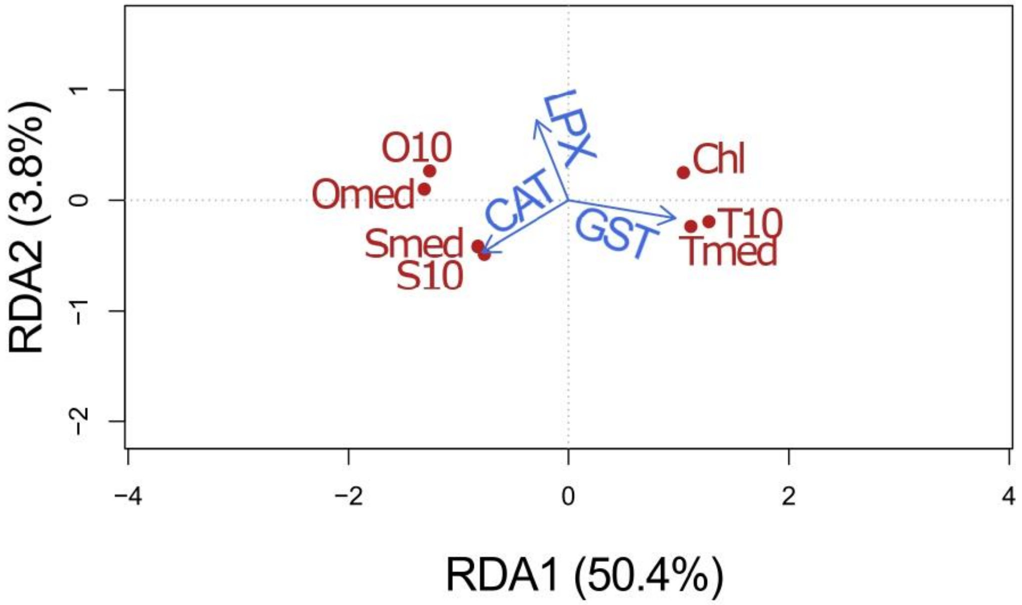

Suppl. Fig 1 RDA biplot showing the relationships between environmental variables—median and 10 m temperature (Tmed, T10), median and 10 m salinity (Smed, S10), median and 10 m dissolved oxygen (Omed, O10) and chlorophyll a (Chl) and biomarker responses, including Catalase activity (CAT), Glutathione-S-transferase activity (GST), and lipid peroxidation (LPX). Blue arrows represent biomarker vectors, and red labels represent environmental variables. Axis 1 (RDA1) explains 50.4% of the variance, and Axis 2 (RDA2) explains 3.8%.

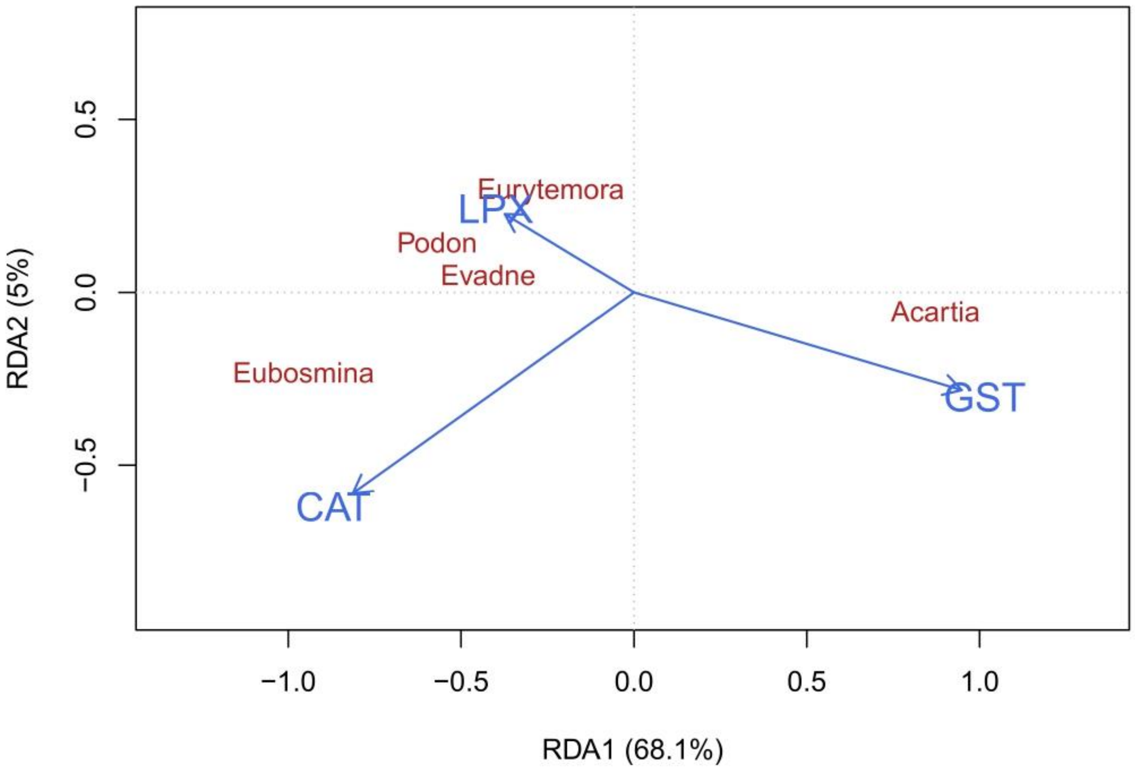

Suppl. Fig. 2 RDA biplot showing the relationships between Acartia spp., Eurytemora affinis, Eubosmina spp., Podon spp., and Evadne spp. and biomarker responses, including Catalase activity (CAT), Glutathione-S-transferase activity (GST), and lipid peroxidation (LPX). Blue arrows represent biomarker vectors, and red labels represent the species variables. Axis 1 (RDA1) explains 68.1% of the variance, and Axis 2 (RDA2) explains 5%.
